# Antiferromagnetic excitonic insulator state in Sr_3_Ir_2_O_7_

**DOI:** 10.1038/s41467-022-28207-w

**Published:** 2022-02-17

**Authors:** D. G. Mazzone, Y. Shen, H. Suwa, G. Fabbris, J. Yang, S.-S. Zhang, H. Miao, J. Sears, Ke Jia, Y. G. Shi, M. H. Upton, D. M. Casa, X. Liu, Jian Liu, C. D. Batista, M. P. M. Dean

**Affiliations:** 1 Department of Condensed Matter Physics and Materials Science, Brookhaven National Laboratory, Upton, NY 11973 USA; 2 Laboratory for Neutron Scattering and Imaging, Paul Scherrer Institut, CH-5232 Villigen, Switzerland; 3 Department of Physics, The University of Tokyo, Tokyo, 113-0033 Japan; 4 Department of Physics and Astronomy, University of Tennessee, Knoxville, TN 37996 USA; 5 Advanced Photon Source, Argonne National Laboratory, Argonne, IL 60439 USA; 6 Materials Science and Technology Division, Oak Ridge National Laboratory, Oak Ridge, TN 37831 USA; 7 Beijing National Laboratory for Condensed Matter Physics, Institute of Physics, Chinese Academy of Sciences, Beijing, 100190 China; 8 School of Physical Science and Technology, ShanghaiTech University, Shanghai, 201210 China; 9 Quantum Condensed Matter Division and Shull-Wollan Center, Oak Ridge National Laboratory, Oak Ridge, TN 37831 USA

**Keywords:** Electronic properties and materials, Magnetic properties and materials

## Abstract

Excitonic insulators are usually considered to form via the condensation of a soft charge mode of bound electron-hole pairs. This, however, presumes that the soft exciton is of spin-singlet character. Early theoretical considerations have also predicted a very distinct scenario, in which the condensation of magnetic excitons results in an antiferromagnetic excitonic insulator state. Here we report resonant inelastic x-ray scattering (RIXS) measurements of Sr_3_Ir_2_O_7_. By isolating the longitudinal component of the spectra, we identify a magnetic mode that is well-defined at the magnetic and structural Brillouin zone centers, but which merges with the electronic continuum in between these high symmetry points and which decays upon heating concurrent with a decrease in the material’s resistivity. We show that a bilayer Hubbard model, in which electron-hole pairs are bound by exchange interactions, consistently explains all the electronic and magnetic properties of Sr_3_Ir_2_O_7_ indicating that this material is a realization of the long-predicted antiferromagnetic excitonic insulator phase.

## Abstract (web summary)

Antiferromagnetic excitonic insulators are a distinct form of excitonic insulator, in which electrons and holes are bound by magnetic exchange rather than Coulomb attraction. Here, Mazzone et al. show, using X-ray scattering, that Sr3Ir2O7 realizes this particular state.

## Introduction

Detailed theoretical considerations of narrow-gap insulators date back to the 1960s, when it was realized that if the energy required to form an electron-hole pair becomes negative, a phase transition into an excitonic insulator state can occur^1–4^. Unscreened electron-hole Coulomb attraction is perhaps the most obvious driving force behind this phase transition, and excitonic charge insulator states are indeed thought to occur in materials such as TmSe_0.45_Te_0.55_, 1T-TiSe_2_, and Ta_2_NiSe_5_^5–9^. Although less intuitive, effective electron-hole attraction can also arise from on-site electron-electron Coulomb repulsion *U* via magnetic exchange interactions between the electron and hole^10^. In this case, the soft exciton is expected to be a spin-triplet, which passes through a quantum critical point (QCP) with increasing effective *U*. The condensation of the relevant triplet exciton at the QCP gives rise to an antiferromagnetic ground state hosting a well-defined excitonic longitudinal mode^4^, which coexists with transverse modes that are a generic feature of ordered antiferromagnets. This longitudinal mode features excitonic character, in the sense that it modifies the local spin amplitude by creating electron-hole pairs^4^. In this work, we identify and study a longitudinal mode in Sr_3_Ir_2_O_7_, the presence of which is the key experimental signature of an antiferromagnetic excitonic insulator.

## Results

**Fig. 1:**
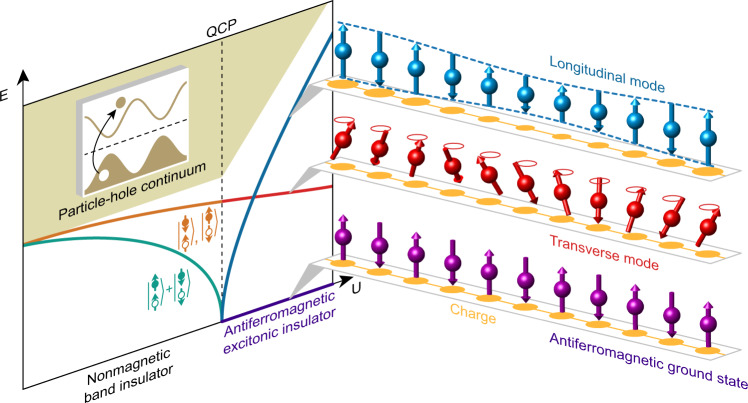
Antiferromagnetic excitonic insulator phase diagram. Charge excitations in paramagnetic band insulators consist of either electron-hole excitations across the insulating band gap (brown shaded area) or of bound electron-hole excitons below the particle-hole continuum [electrons (holes) are indicated with filled (empty) circles]. An antiferromagnetic excitonic insulator is established through the condensation of the predominately spin-triplet character exciton mode with spin quantum number *S*^*z*^ = 0. The excition is a superposition of an up-spin electron in the conduction band paired with an up-spin hole (equivalent to a down-spin electron) and a down-spin electron paired with a down-spin hole^4^. The other spin-triplet excitions *S*^*z*^ = ±1 feature an up-spin electron and a down-spin hole or a down-spin electron and an up-spin hole. Upon increasing Coulomb interaction *U* the *S*^*z*^ = 0 exciton condenses into the ground state at a QCP^4^, establishing magnetic order and leaving an excitonic longitudinal mode as the key signature of this state.

**Fig. 2:**
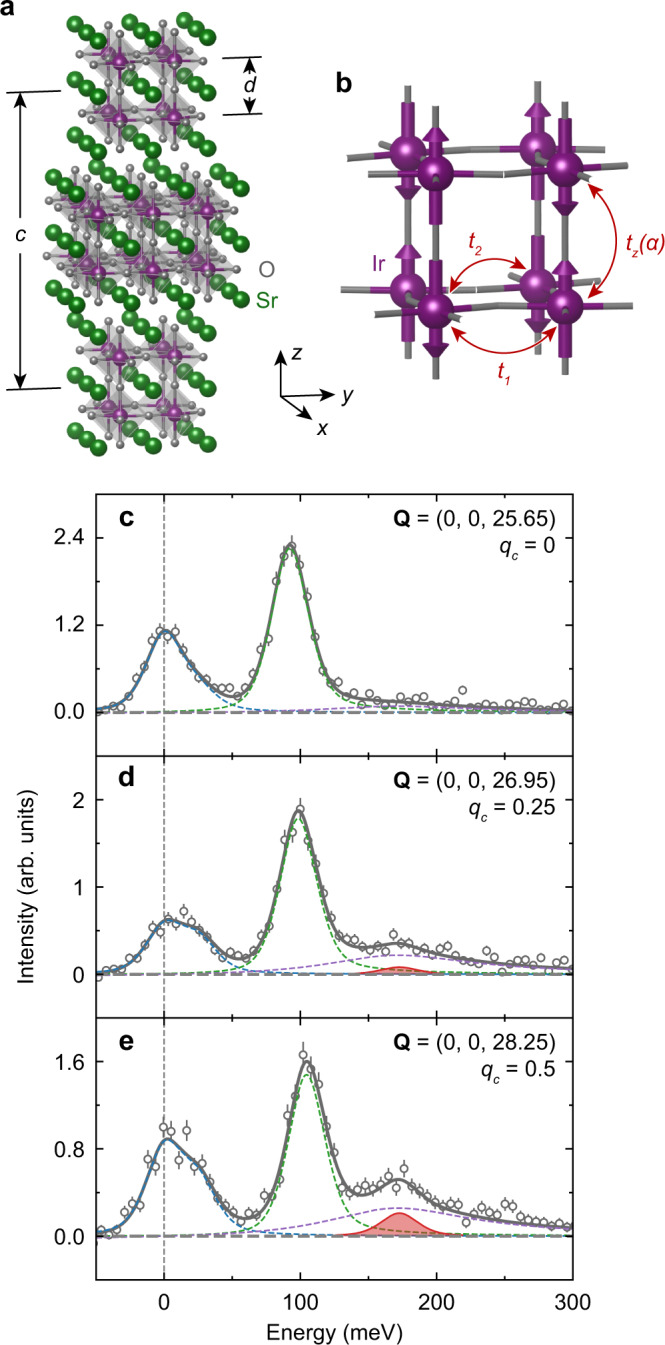
Isolating the excitonic longitudinal mode in Sr_3_Ir_2_O_7_. **a** Crystal structure of the bilayer material Sr_3_Ir_2_O_7_. **b** Ir-Ir bilayer with *t*_1_ the nearest-neighbor, *t*_2_ the next-nearest-neighbor and *t*_*z*_(*α*) the interlayer hopping terms. **c**–**e** RIXS spectra measured at *T* = 20 K and **Q** = (0, 0, *L*) with *L* = 25.65, 26.95 and 28.25 in reciprocal lattice units. The *c*-axis positions are also labeled in terms of the Ir-Ir interlayer reciprocal-lattice spacing *q*_*c*_ = 0, 0.25 and 0.5. An additional mode appears around 170 meV with maximal intensity at *q*_*c*_ = 0.5 (see shaded red area). The black circles represent the data and dotted lines outline the different components of the spectrum, which are summed to produce the grey line representing the total spectrum. Error bars are determined via Poissonian statistics.

The formation of an antiferromagnetic excitonic insulator requires a very specific set of conditions. We need (i) a charge gap of similar magnitude to its magnetic energy scale and (ii) strong easy-axis anisotropy. Property (i) is a sign that the material is close to the excitonic QCP (see Fig. 1). Property (ii) is not a strict condition, but it facilitates the identification of an antiferromagnetic excitonic insulator, because the opening of a spin gap Δ_*s*_ protects the longitudinal mode from decay. This is because longitudinal fluctuations are often kinetically predisposed to decay into transverse modes generating a longitudinal continuum with no well-defined modes. This decay can be avoided when the energy of the longitudinal mode is lower than twice the spin gap. Iridates host strong spin-orbit coupling (SOC), which can help realize a large spin gap and bilayer Sr_3_Ir_2_O_7_ shown in Fig. 2a is known to have a narrow charge gap of order Δ_*c*_ ~ 150 meV^11^. The essential magnetic unit, with *c*-axis ordered moments, is shown in Fig. 2b^12^. In view of the antiferromagnetic order in Sr_3_Ir_2_O_7_, the material would be predicted to lie in the magnetically ordered region to the right of the QCP where the excitonic longitudinal mode is expected to appear. Because the exciton is predicted to have odd parity under exchange of the two Ir layers, we expect the excitonic longitudinal mode to be present at *c*-axis wavevectors corresponding to antisymmetric bilayer contributions and absent at the symmetric condition. We label these wavevectors *q*_c_ = 0.5 and *q*_c_ = 0, respectively. In contrast, transverse magnetic modes are expected to be present at all *c*-axis wavevectors, allowing the transverse and longitudinal modes to be readily distinguished.

The excitation spectrum of Sr_3_Ir_2_O_7_ was studied with RIXS. Figure 2c–e displays energy-loss spectra at *T* = 20 K, well below the Néel temperature *T*_N_ = 285 K and *q*_c_ = 0, 0.25 and 0.5, corresponding to *L* = 25.65, 26.95 and 28.25 in reciprocal lattice units (r.l.u.). These irrational *L* values arise because the bilayer separation *d* is not a rational fraction of the unit cell height *c* (see Methods section for details). The spectrum at *q*_*c*_ = 0 is composed of a phonon-decorated quasi-elastic feature, a pronounced magnetic excitation at ~ 100 meV, which we later identified as the transverse mode, and a high-energy continuum. As explained above, changing *q*_*c*_ is expected to isolate the anticipated excitonic mode. A longitudinal mode is indeed observed, reaching maximum intensity at *q*_*c*_ = 0.5, and is highlighted by red shading in Fig. 2d, e.

**Fig. 3:**
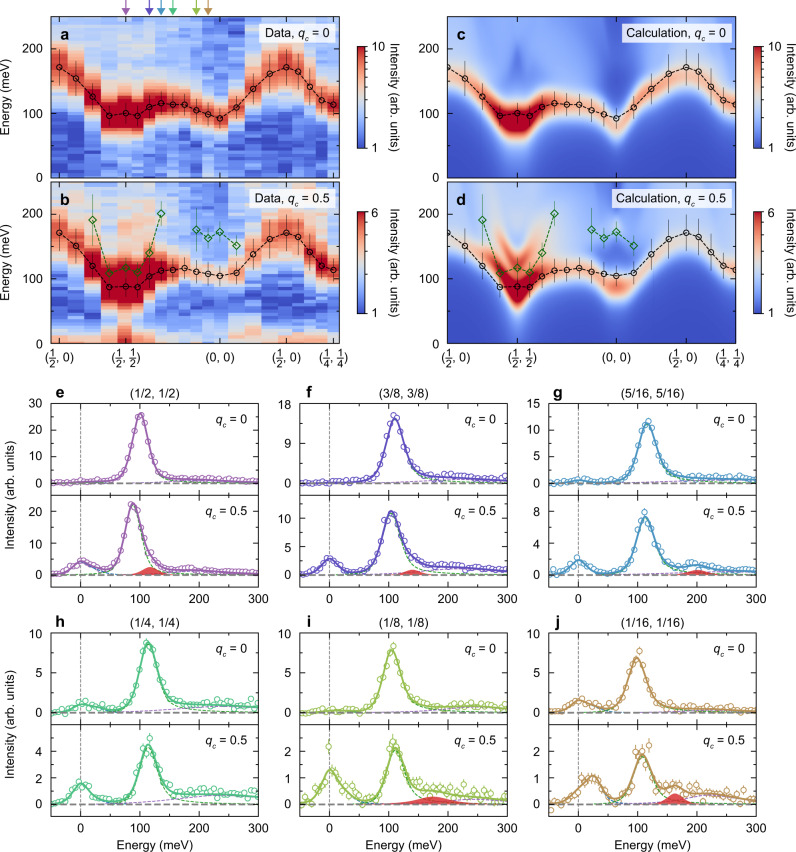
Magnetic dispersion and excitonic longitudinal mode decay. **a**, **b** In-plane momentum dependence of the magnetic excitations measured at *q*_*c*_ = 0 and 0.5. The black and green symbols correspond to the energy of the magnetic modes and the vertical bars to their peak widths. Both quantities were extracted from the energy spectra at different points in reciprocal space (such as shown in panels **e–j** and Fig. 2c–e). **c** and **d** Theoretical calculations of the magnetic dispersion relation, overplotted with the experimentally determined excitation energies and line widths. The presence of the mode at *q*_*c*_ = 0.5 that is absent at *q*_*c*_ = 0 evinces that this is an excitonic longitudinal mode. **e**–**j** RIXS spectra at reciprocal space as highlighted by color-matching arrows in panel **a**. Circles represent the data and dotted lines outline the different components of the spectrum, which are summed to produce the solid line representing the total spectrum. Error bars are determined via Poissonian statistics. The isolation of the longitudinal mode (highlighted with red shading) from other contributions was possible by simultaneously analyzing *q*_*c*_ = 0.5 and *q*_*c*_ = 0 for each in-plane reciprocal-lattice wavevector (see Methods section for details).

In isolation, the presence of a longitudinal magnetic mode in this symmetry channel is a necessary but insufficient condition to establish an antiferromagnetic excitonic insulator, so we leverage the specific symmetry, decay, and temperature dependence of the longitudinal and transverse magnetic modes to establish the presence of the novel state. The only other candidate magnetic model that hosts a longitudinal mode of this type is a specific configuration of the bilayer Heisenberg Hamiltonian, in which the charge degrees of freedom are projected out. In particular, a model with a *c*-axis magnetic exchange *J*_*c*_ that is larger than, but not dramatically larger than, the in-plane exchange *J*_*ab*_ is needed to produce a longitudinal mode and large easy-axis magnetic anisotropy is required to reproduce the spin gap. If *J*_*c*_ ≪ *J*_*ab*_, the spectrum would show only a spin-wave-like in-plane dispersion contrary to the observed *q*_*c*_ dependence in Fig. 2c–e, and in the *J*_*c*_ ≫ *J*_*ab*_ limit the system would become a quantum paramagnet. For *J*_*c*_/*J*_*ab*_ of order two, the bilayer Heisenberg Hamiltonian supports a longitudinal mode and for the current case of large easy-axis anisotropy, the transverse and longitudinal modes appear as well-defined modes throughout the Brillouin zone^13–16^. In fact, earlier reports have proposed this spin dimer model to explain RIXS measurements of the longitudinal ~ 170 meV feature in Sr_3_Ir_2_O_7_^14, 17^. Although prior and subsequent non-dimerized models have also been proposed to describe Sr_3_Ir_2_O_7_ as rival candidates^12, 18–21^. These models, however, do not support a longitudinal mode (a detailed comparison between the different models is given in Supplementary Information (SI) Section 1). We, therefore, map the in-plane dispersion relations at *q*_*c*_ = 0 and 0.5 and show them in Fig. 3a, b. At *q*_*c*_ = 0, where the longitudinal mode is suppressed by symmetry, we observe an excitation dispersing from ~ 90 to 170 meV and a continuum at higher energies. Simultaneously analyzing *q*_*c*_ = 0.5 and *q*_*c*_ = 0 for each in-plane reciprocal-lattice wavevector, while leveraging the distinct symmetry properties of the longitudinal and transverse modes, allows us to isolate the longitudinal mode (see Methods section). We plot the position and peak width of the longitudinal mode in green in Fig. 3b. The transverse mode, on the other hand, is symmetry-allowed at *q*_*c*_ = 0.5 and *q*_*c*_ = 0 and is shown in black on Fig. 3a, b. We find that the longitudinal mode is well-defined around (0, 0) (Figs. 2c–d,  3i), but decays into the high-energy continuum as it disperses away, becoming undetectable at (1/4, 1/4) (Fig. 3h). The longitudinal mode is also detectable as a shoulder feature on the transverse mode at (1/2, 1/2) before dispersing upwards and broadening at neighboring momenta (Fig. 3e, g). The decay and merging of the longitudinal mode into the electron-hole continuum was not detected previously and suggests the realization of an antiferromagnetic excitonic insulator state because the longitudinal mode in this model has a bound electron-hole pair character and therefore will necessarily decay when it overlaps with the electron-hole continuum. This longitudinal mode decay is incompatible with a longitudinal mode arising from spin dimer excitations in a strongly isotropic bilayer Heisenberg model, which predicts well-defined modes throughout the Brillouin zone and projects out the high-energy particle-hole continuum^13–16^.

Since optical conductivity, tunneling spectroscopy, and photo-emission studies all report charge gaps Δ_*c*_ on the same energy scale of magnetic excitations (100–200 meV)^11, 22–25^, we model the microscopic interactions within a Hubbard Hamiltonian that retains the charge degree of freedom. In particular, the crucial difference with the Heisenberg description is that the Hubbard model retains the electron-hole continuum, whose lower edge at *ω* = Δ_*c*_ is below the onset of the two-magnon continuum: Δ_*c*_ < 2Δ_*s*_. We considered a half-filled bilayer, which includes a single “*J*_eff_ = 1/2” effective orbital for each of the two Ir sites in the unit cell, following methods developed in parallel with this experimental study^26^. The model contains an effective Coulomb repulsion *U*, and three electron hopping parameters: nearest and next-nearest in-plane hopping terms *t*_*ν*_ (*ν* = 1, 2) within each Ir layer, and the spin dependent hopping strength *t*_*z*_(*α*) between Ir layers (Fig. 2b). *t*_*z*_(*α*) is composed of an amplitude ∣*t*_*z*_∣ and a phase *α* arising from the appreciable SOC in the material (further details are given in the Methods section)^27^. The model was solved using the random phase approximation (RPA) in the thermodynamic limit (SI Section 2), which is valid for intermediately correlated materials even at finite temperature^28^. We constrain *t*_*ν*_ and ∣*t*_*z*_∣ to values compatible with density functional theory and photo-emission measurements and consider the effective *U*, which is strongly influenced by screening, as the primary tuning parameter^29^. Figure 3c, d show the results of calculations with *t*_1_ = 0.115 eV, *t*_2_ = 0.012 eV, ∣*t*_*z*_∣ = 0.084 eV, *α* = 1.41, and *U* = 0.325 eV. The small *U* is due to the extended Ir orbitals and because this effective parameterization reflects the difference between on-site and longer-range interactions in the real material. The model identifies the quasiparticle dispersion at *q*_*c*_ = 0 as the transverse mode with a persistent well-defined nature even at high energies. Above the transverse mode, the spin response is fundamentally influenced by the finite charge gap. A broad continuum involving electron-hole spin transitions across the charge gap is present for all *q*_*c*_ values covering a broad energy-momentum range. A new mode emerges around (0, 0) and (0.5, 0.5) for *q*_*c*_ = 0.5, which we identify as the excitonic longitudinal mode.

To understand the excitonic longitudinal mode discussed, we first note that the tight-binding band structure analysis of Sr_3_Ir_2_O_7_ suggests that it would be a narrow-gap band insulator or semi-metal even when Coulomb repulsion is neglected^29^. This occurs due to bonding-antibonding band splitting arising from the bilayer hopping alongside SOC, generating a minimum of the conduction band dispersion near the Brillouin zone center and a maximum in the valence band dispersion near the antiferromagnetic zone center. A finite value of *U* in a quasi-two-dimensional bilayer structure such as Sr_3_Ir_2_O_7_ produces an attractive particle-hole interaction in the triplet channel because of the well-known direct-exchange mechanism. In turn, particle-hole pairs at wavevectors favored by the band structure form bound states, i.e., excitons, in the magnetic channel appearing at *q*_*c*_ = 0.5, because of the odd parity of the exciton under exchange of the two layers. The spin anisotropy arising from SOC splits the exciton triplet into a low energy state with *c*-axis spin quantum number *S*^*z*^ = 0 and higher energy *S*^*z*^ = ± 1 states. Strictly speaking, SOC means that total spin is not a good quantum number, but we retain the singlet-triplet labels for clarity. As shown in the schematic representation in Fig. 1, the *S*^*z*^ = 0 exciton condenses to form magnetic order at a wavevector of (0.5, 0.5) (*q*_*c*_ = 0.5). The corresponding QCP, which exists at *U* = *U*_c_ = 0.27 eV (for *t*_1_ = 0.115 eV), then signals the onset of the antiferromagnetic excitonic insulator state in Sr_3_Ir_2_O_7_. Within the ordered state, what was a gapless *S*^*z*^ = 0 exciton mode at *U* = *U*_*c*_ becomes a gapped excitonic longitudinal mode for *U* > *U*_*c*_. The existence and relatively low energy of this mode implies that *U* in Sr_3_Ir_2_O_7_ is only slightly above *U*_*c*_. This property, together with the sufficiently large transverse mode gap Δ_*s*_, protects the excitonic longitudinal mode from decay into pairs of transverse modes. The longitudinal mode’s bound electron-hole pair nature is especially vividly illustrated by its smooth merging with the particle-hole continuum away from (0.5, 0.5) and (0, 0). We plot the layer-resolved charge structure of the exciton in SI Section 3.

**Fig. 4:**
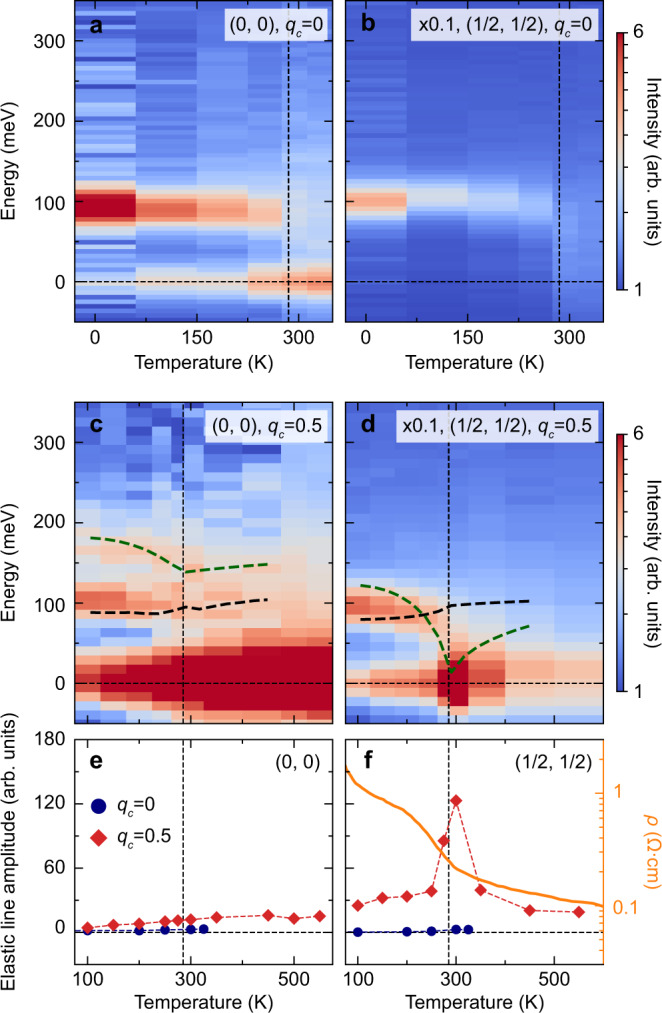
Excitonic mode condensation at the Néel temperature. **a**–**d** Temperature dependence of the Sr_3_Ir_2_O_7_ excitation spectrum at (0, 0) and (0.5, 0.5) for *q*_*c*_ = 0 and 0.5 (RIXS spectra at selected temperatures are shown in Supplementary Fig. S2). The intensity at (0.5, 0.5) has been scaled for comparison reasons. The dashed lines show temperature-dependent calculations of our model (the full theoretical predictions are plotted in Supplementary Fig. S5). Based on the *q*_*c*_ behavior of the modes, we know that panels **a**, **b** show only the transverse mode, while **c**, **d** show both the transverse and longitudinal mode. **e**, **f** Quasi-elastic intensity as function of temperature for *q*_*c*_ = 0 and 0.5 in blue and red, respectively. The non-monotonic enhancement at *q*_*c*_ = 0.5 in **e** provides additional support that the condensation of the excitonic longitudinal mode establishes the magnetic long-range order in Sr_3_Ir_2_O_7_. Panel **f** also shows the anomalous temperature dependence of the electric resisitivity *ρ* (taken from^30^), which shows a change in gradient at *T*_N_ further indicating that charge fluctuations are involved in the transition.

When heating an antiferromagnetic excitonic insulator, thermal fluctuations modify the magnetic properties via two different processes. The first one corresponds to the destruction of Néel order via softening of the longitudinal mode. This softening signals the exciton condensation below *T* = *T*_*N*_. The second process, that takes place at a higher temperature T^*^, corresponds to thermal breaking of the excitons (unbinding of particle-hole pairs). A RIXS temperature series designed to test this idea at different high symmetry locations is plotted in Fig. 4a–d (linecuts at selected temperatures are shown in Supplementary Fig. S2). As expected, heating up from base temperature towards *T*_N_ enhances the decay of the modes into the electron-hole continuum broadening the spectra and making it difficult to isolate the two modes in a single spectrum. We can, however, leverage the symmetry properties of the modes at different reciprocal space points to clarify the soft mode phenomenology. Since the transverse mode occurs at the same energy independent of *q*_*c*_, and the longitudinal mode is present at *q*_*c*_ = 0.5 and absent at *q*_*c*_ = 0, the transverse mode temperature dependence can be studied in isolation at *q*_*c*_ = 0 (Fig. 4a, b). We observe that this mode has only minimal detectable softening, which is expected in view of the Ising nature of magnetism. In contrast, a substantial softening is seen at (0.5, 0.5) in Fig. 4d. Although both modes are present at *q*_*c*_ = 0.5, we know from *q*_*c*_ = 0 measurements that the transverse mode displays only minimal softening. Thus the longitudinal mode must play a major role in the softening to form the antiferromagnetic state. Our observed phenomenology is only captured with the intermediate coupling regime (*U*/*t*_1_ = 2.83) that we conclude is relevant for Sr_3_Ir_2_O_7_. The strong coupling limit (*U*/*t*_1_ ≫ 1) would require a charge gap much larger than the observed values of 100–200 meV and, to our knowledge, it has not been able to predict any aspects of the temperature-dependent phenomenology of Sr_3_Ir_2_O_7_. The excitonic insulator model is also supported by our temperature-dependent calculations, which are shown as dashed lines in Fig. 4c, d. Full calculations are shown in Supplementary Fig. S5 and explained in SI Section 2. Theory shows that exciton formation takes place at T^*^ ≈ 2*T*_N_, controlled by the exciton binding energy, which is of order the charge gap minus the longitudinal mode energy at the ordering wavevector. The mean-field transition temperature prediction is *T*_N_ = 424 K, which is not too far above the measured *T*_N_ = 285 K and which is expected since fluctuations are expected to reduce *T*_N_ below the mean-field prediction. The predictions in Fig. 4c, d are shown with temperatures re-normalized to the experimental *T*_N_.

The involvement of the longitudinal mode in magnetic long-range order is also evident from the temperature dependent quasi-elastic intensity. While most spectra feature the expected gradual enhancement in the quasi-elastic channel upon increasing temperature (Fig. 4e for (0, 0) and S2 for other reciprocal-lattice positions), the (0.5, 0.5) spectrum at *q*_*c*_ = 0.5 displays a pronounced rise of intensity around *T*_N_ (Fig. 4f). Note that neither *q*_*c*_ = 0 nor *q*_*c*_ = 0.5 correspond to the magnetic Bragg peak location, because the bilayer separation is incommensurate with respect to the *c*-axis lattice constant. Since in our setup *q*_*c*_ = 0 is closer to a magnetic Bragg peak than *q*_*c*_ = 0.5, we can exclude critical scattering from the long-range antiferromagnetic order as a significant contributor to this intensity as it would predict the opposite intensity behavior to what we observe (a more extensive demonstration of this is in SI Section 5). Thus the observed quasi-elastic anomaly at *T*_N_ is indicative of substantial longitudinal mode condensation. The excitonic insulator character of the ground state is further supported by a large increase in resistivity below *T*_N_ (see Fig. 4f)^30^, as the condensation of the excitonic mode leads to a reduction in the electronic carriers participating in electrical transport. This property is distinct from what is expected for a strongly-coupled Mott insulator (i.e., the large *U* limit of Fig. 1) where all charge-related processes are frozen out. The resistivity increase below *T*_N_ could, in principle, also arise from Slater-type interactions, which can open a charge gap upon magnetic ordering. Sr_3_Ir_2_O_7_, however, lacks strong Fermi surface nesting^23–25, 29^ and is in the intermediately correlated (*t*_1_ ~ *U*) rather than the weakly correlated (*t*_1_ ≫ *U*) regime, so the Slater mechanism is expected to have minimal relevance.

## Discussion

In summary, we have isolated and characterized a longitudinal magnetic mode in Sr_3_Ir_2_O_7_, which merges with the electron-hole continuum at certain points in the Brillouin zone, and which softens upon heating concurrent with a decrease in the material’s resistivity. These properties are consistent with those of an antiferromagnetic excitonic insulator state^4^. We substantiate this via calculations of a bilayer Hubbard model, in which electron-hole pairs are bound by magnetic exchange interactions between the electron and hole. This consistently explains all the electronic and magnetic properties of Sr_3_Ir_2_O_7_ based on only one free parameter *U*, since all other parameters are strongly constrained by the electronic band structure of the material. The totality of these results identifies Sr_3_Ir_2_O_7_ as a compelling candidate for the long-sought-after antiferromagnetic excitonic insulator.

Looking to the future, the intrinsically coupled spin and charge degrees of freedom in this state could have the potential for realizing new functionalities^31^, and suitably tuned material and/or laser-based approaches could realize methods to photo-excited these modes^32^. Further research on the topic may also include efforts to identify materials closer to the QCP, which in our study occurs at *U*/*t*_1_ = 2.35. This could extend the reciprocal space regions where the excitonic longitudinal mode exists. Another interesting direction would involve identifying excitonic easy-plane, rather than easy-axis, bilayer systems. These would host a different kind of soft excitonic longitudinal mode, often called “Higgs” mode, and could be used to study Higgs decay and renormalization effects in the presence of strong charge fluctuations. Careful selection of materials with multiple active orbitals could realize orbitally-ordered excitonic insulator states. Experimental realizations using chemical substitutions, strained thin films, high pressure, or different bilayer materials, including ruthenates, osmates, and other iridates, may help to answer some of these intriguing questions.

## Methods

### Samples

Sr_3_Ir_2_O_7_ single crystals were synthesized using the flux method^33^. Starting materials of IrO_2_, SrCO_3_, and SrCl_2_ ⋅ 6H_2_O were mixed with a molar ratio of 1:2:20, and heated at 1200 ^∘^C for 10 h in a platinum crucible. The melt was then cooled to 800°C at a rate of 3 ^∘^C/h, before quenching to room temperature. We index reciprocal space using a pseudo-tetragonal unit cell with *a* = *b* = 3.896 Å and *c* = 20.88 Å at room temperature.

### Resonant inelastic X-ray scattering (RIXS) setup

RIXS spectra were measured at the 27-ID-B station of the Advanced Photon Source at Argonne National Laboratory. The incident x-ray beam was tuned to the Ir *L*_3_-edge at 11.215 keV and monochromated using a Si (884) channel-cut monochromator. The exact x-ray energy was refined via resonant energy of a standard IrO_2_ and the Sr_3_Ir_2_O_7_ sample and was set 3 eV below the resonant edge. Scattered photons were analyzed using a spherically bent diced silicon (844) analyzer with a curvature radius of 2 m. The energy and Q-resolution were 32.0(2) meV and 0.105 Å^−1^ full-width at half-maximum (FWHM), respectively. A small background contribution arising from air scattering was removed by subtracting a constant value from the measured intensity. The value was determined by fitting the intensity on the energy-gain side of the spectra.

The *L* values in Fig. 2c–e were chosen such that they correspond to specific reciprocal-lattice positions with respect to the Ir-Ir interlayer spacing (see also Fig. 2a), i.e., *G* + *q*_*c*_ = *Ld*/*c*, where *G* is an integer, *q*_*c*_ the reduced *c*-axis reciprocal lattice position in terms of the Ir-Ir spacing, *d* = 4.07 Å the shortest Ir-Ir interlayer spacing and *c* = 20.88 Å the out-of-plane lattice constant. *q*_*c*_ equals 0, 0.25 and 0.5 for *L* = 25.65, 26.95 and 28.25, respectively.

The magnetic dispersions in Fig. 3a, b were measured along (*H*_1_, *K*_1_, 25.65) and (*H*_2_, *K*_2_, 28.25) with *H*_1_ and *K*_1_ ranging between 0.5 and 1 and *H*_2_ and *K*_2_ between 0 and 0.5. The particular Brillouin zones were chosen to ensure a scattering geometry close to 90^∘^, minimizing Thompson scattering. For (0, 0, 25.65), (1, 1, 25.65), (0, 0, 26.92) and (0, 0, 28.25), 2*θ* = 85.5, 90.2, 90.9 and 96.8^∘^, respectively. The sample was aligned in the horizontal (*H*, *H*, *L*) scattering plane, such that both dispersions could be probed through a sample rotation of Δ*χ* ≤ 4.1^∘^ relative to the surface normal.

### Analysis of the RIXS data

The spectra were analyzed by decomposing them into four components: (1) A quasi-elastic contribution (possibly containing contributions from phonons) which was modeled using a pseudo-Voigt energy resolution function, along with an additional low energy feature, which was modeled using the resolution functions at ± 32 meV, whose relative weights were constrained to follow the Bose factor. (2) The transverse magnetic mode was accounted for by a pseudo-Voigt function multiplied with an error function to capture the high-energy tail arising from the interactions with continuum. The interactions are enhanced when the modes and the continuum are less separated in energy, which leads to a reduced quasiparticle lifetime. In this case, we used a damped harmonic oscillator (with Bose factor) that was convoluted with the resolution function, which was further multiplied by an error function. (3) The longitudinal mode was described by either a pseudo-Voigt function or a damped harmonic oscillator, depending on whether or not it was resolution limited. (4) The magnetic continuum was reproduced using a broad damped harmonic oscillator multiplied by an error function to mimic its onset.

The excitonic longitudinal mode is strongly *q*_*c*_ dependent, whereas the transverse magnetic mode and the magnetic continuum vary very weakly with *q*_*c*_. Thus, we analyzed the spectra measured at *q*_*c*_ = 0 and *q*_*c*_ = 0.5 simultaneously to disentangle the excitonic contribution from the other components. The positions and lineshapes of the transverse magnetic mode and the electron-hole magnetic continuum were constrained to be independent of *q*_*c*_, i.e., only the amplitudes were varied. The extra peaks at *q*_*c*_ = 0.5 give information about the excitonic longitudinal mode. During the procedure, the elastic energy was allowed to vary to correct for small fluctuations of the incident energy.

### Theoretical model

Sr_3_Ir_2_O_7_ hosts Ir^4+^ ions, which have 5 electrons in the active Ir 5*d*^5^ valance band. The dominant splitting of this band comes from the close-to-cubic crystal field leaving empty *e*_*g*_ states and 5 electrons in the *t*_2*g*_ states. SOC further splits the *t*_2*g*_ manifold into a full *J*_eff_ = 3/2 orbital a half-filled *J*_eff_ = 1/2 orbital at the Fermi level^34^. Our model involves projecting the band structure onto this *J*_eff_ = 1/2 doublet. The basic structural unit, shown in Fig. 2b, contains two Ir atoms, so the experimental data were interpreted using a half-filled bilayer Hubbard model *H* = − *H*_K_ + *H*_I_ with *H*_I_ = *U*∑_***r***_*n*_***r****↑*_*n*_***r****↓*_ and

1 $${H}_{{{{{{{{\rm{K}}}}}}}}}=\mathop{\sum}\limits_{{{{{{{{\boldsymbol{r}}}}}}}},{{{{{{{{\boldsymbol{\delta }}}}}}}}}_{\nu }}{t}_{\nu }{c}_{{{{{{{{\boldsymbol{r}}}}}}}}}^{{{{\dagger}}} }{c}_{{{{{{{{\boldsymbol{r}}}}}}}}+{{{{{{{{\boldsymbol{\delta }}}}}}}}}_{\nu }}+\mathop{\sum}\limits_{{{{{{{{{\boldsymbol{r}}}}}}}}}_{\perp }}{c}_{({{{{{{{{\boldsymbol{r}}}}}}}}}_{\perp },1)}^{{{{\dagger}}} }{t}_{z}(\alpha ){c}_{({{{{{{{{\boldsymbol{r}}}}}}}}}_{\perp },2)}+{{{{{{{\rm{H}}}}}}}}.{{{{{{{\rm{c}}}}}}}}.,$$

where *t*_*ν*_ (*ν* = 1, 2) are the nearest- and next-nearest-neighbor hopping amplitudes within the square lattice of each Ir-layer, and ${t}_{z}(\alpha )=| {t}_{z}| {e}^{i\frac{\alpha }{2}{\varepsilon }_{{{{{{{{\boldsymbol{r}}}}}}}}}{\sigma }_{z}}$, with *σ*_*z*_ the Pauli matrix describes the *J*_eff_ spin dependent hopping strength between layers. The overall phase was chosen to gauge away the phase for *t*_*ν*_. The operator ${c}_{{{{{{{{\boldsymbol{r}}}}}}}}}^{{{{\dagger}}} }$ = [${c}_{\uparrow ,{{{{{{{\boldsymbol{r}}}}}}}}}^{{{{\dagger}}} }$, ${c}_{\downarrow ,{{{{{{{\boldsymbol{r}}}}}}}}}^{{{{\dagger}}} }$] creates the Nambu spinor of the electron field at ***r*** = (***r***_⊥_, *l*) with *l* = 1, 2 denoting the layer index and ***r***_⊥_ = *r*_1_***a***_1_ + *r*_2_***a***_2_. Here, the primitive in-plane lattice vectors are denoted by ***a***_1_ and ***a***_2_, and the directed neighboring bonds are represented by ***δ***_1_ = ***a***_1_, ***a***_2_ and ***δ***_2_ = ***a***_1_ ± ***a***_2_. In the interaction term *H*_I_, *U* is the effective Coulomb interaction, and *n*_***r****σ*_ is the density operator for electrons of spin *σ* at ***r***. In the spin dependent hopping term, the sign *ε*_***r***_ takes the values ± 1 depending on which sublattice of the bipartite bilayer system ***r*** points to. The phase *α* arises from hopping matrix elements between *d*_*xz*_ and *d*_*yz*_ orbitals, which are allowed through the staggered octahedral rotations in the unit cell along side SOC^27, 35^. In the model, SOC enters via the phase of the *c*-axis hopping, which is smaller than the in-plane bandwidth, justifying the approximate use of singlet and triplet for labels of the different excitons. The model was studied at half-filling in the sense that it contains two bands (bonding and antibonding) in the model, which host two electrons as is appropriate for Sr_3_Ir_2_O_7_^23–25, 27^. We solved the model using the RPA in the thermodynamic limit (detailed information is given in SI Section 2), which is valid for intermediately correlated materials even at finite temperatures^28^. The theoretically determined Néel temperature, in this case, is ${T}_{\,{{\mbox{N}}}}^{{{\mbox{cal}}}\,}=424$ K which is slightly larger than the experimental value *T*_N_ = 285 K. This is expected within the RPA we use here, as this ignores fluctuations than act to reduce the transition temperature. The dynamical spin structure factors in Fig. 3c, d are shown after convolution with the experimental resolution.

A more complex model could include all *t*_2*g*_ or all *d* orbitals, rather than just effective *J*_eff_ = 1/2 doublets. The success of our *J*_eff_ = 1/2 only model suggests that orbital degrees of freedom are entirely frozen out of the problem or manifest themselves in very subtle ways beyond current detection limits. Due to this, the Sr_3_Ir_2_O_7_ excitonic insulator state has no orbital component (other than in the trivial sense that the *J*_eff_ = 1/2 states in themselves are a coupled modulation of spin and orbital angular momentum). A possible SOC-induced orbital order is discussed in SI Section 6.

## Supplementary information

Supplementary Information

## Data Availability

The RIXS data generated in this study have been deposited in the Zenodo database under accession code 5812989^36^.

## Code availability

The code used in this study is available from the authors upon reasonable request.
