# Supplementary material for: Antiferromagnetic excitonic insulator state in Sr3Ir2O7
Source: Nat Commun. 2022 Feb 17;13:913. doi: 10.1038/s41467-022-28207-w (PMC8854623; doi:10.1038/s41467-022-28207-w)
Supplement: Supplementary file 1 — Supplementary Information [file 41467_2022_28207_MOESM1_ESM.pdf]

Antiferromagnetic Excitonic Insulator State in $Sr_3Ir_2O_7$
Supplementary Information

D. G. Mazzone,[1,2,*] Y. Shen,[1] H. Suwa,[3,4] G. Fabbris,[5] J. Yang,[4] S-S. Zhang,[4] H. Miao,[6] J. Sears,[1] Ke Jia,[7] Y. G. Shi,[7] M .H. Upton,[5] D. M. Casa,[5] X. Liu,[8,†] J. Liu,[4] C. Batista,[4,9] and M. P. M. Dean[1,‡]

[1] *Department of Condensed Matter Physics and Materials Science, Brookhaven National Laboratory, Upton, New York 11973, USA*
[2] *Laboratory for Neutron Scattering and Imaging, Paul Scherrer Institut, CH-5232 Villigen, Switzerland*
[3] *Department of Physics, The University of Tokyo, Tokyo 113-0033, Japan*
[4] *Department of Physics and Astronomy, University of Tennessee, Knoxville, Tennessee 37996, USA*
[5] *Advanced Photon Source, Argonne National Laboratory, Argonne, Illinois 60439, USA*
[6] *Material Science and Technology Division, Oak Ridge National Laboratory, Oak Ridge, Tennessee 37831, USA*
[7] *Beijing National Laboratory for Condensed Matter Physics, Institute of Physics, Chinese Academy of Sciences, Beijing 100190, China*
[8] *School of Physical Science and Technology, ShanghaiTech University, Shanghai 201210, China*
[9] *Quantum Condensed Matter Division and Shull-Wollan Center, Oak Ridge National Laboratory, Oak Ridge, TN 37831, USA*

CONTENTS

1. Other models for magnetism in $Sr_3Ir_2O_7$ 1

2. Mean field and random phase approximations 2

3. Illustration of the exciton 4

4. Excitonic longitudinal mode condensation at T_N 4

5. Minimal contribution of critical scattering to quasi-elastic intensity 8

6. Orbital currents 9

References 9

1. OTHER MODELS FOR MAGNETISM IN $Sr_3Ir_2O_7$

Previous work on $Sr_3Ir_2O_7$ described the magnetic properties of the material using Heisenberg models, which project out any active charge degrees of freedom [1–5]. The magnetic dispersion was then calculated either under the linear spin-wave approximation or using bond-operator theory for a square lattice of dimers [1, 2, 4]. Although both models can be refined to reproduce features of the experimental spectra, it is notable that large numbers of unconstrained parameter are required and that both approaches lead to complications.

The spin-wave treatment predicts a spectrum composed of two transverse optical and acoustic magnetic excitations with energies that should be equal upon reflection about the magnetic zone boundary, which is hard to reconcile with the measured spectra [1, 2]. In addition, multiple higher-order couplings that are barely weaker than the nearest neighbour interactions are required. These terms suggest that the $U/t_1 \gg 1$ limit, in which projecting the Hubbard model onto the Heisenberg model is valid, is not appropriate for $Sr_3Ir_2O_7$ [6, 7]. Further, the model cannot account for the additional magnetic intensity at $q_c = 0.5$, as physics beyond linear spin-wave theory is needed to model longitudinal quasiparticles. If spin-wave theory is extended to include a longitudinal response, a continuum and not an additional mode would be generated. More recently, a Raman scattering study proposed a spin-wave-theory based

* daniel.mazzone@psi.ch
† liuxr@shanghaitec.edu.cn
‡ mdean@bnl.gov

model for the magnetic excitations in $Sr_3Ir_2O_7$ [5], but uses very different parameters to both of the other models and is inconsistent with both resonant inelastic x-ray scattering (RIXS) and inelastic neutron scattering spectra [8].

The dimer approach does yield a longitudinal mode and a transverse mode, but it fails to predict the decay of the longitudinal mode away from $(0, 0)$ and $(0.5, 0.5)$. Another issue with this approach is that it requires magnetic interactions that are hard to physically justify as the ratio between the c-axis and ab-plane magnetic interactions should be much greater than one for this model to be valid, and to adequately reproduce the data [2, 4]. This is questionable because the in-plane Ir-Ir bonds in $Sr_3Ir_2O_7$ are actually shorter than the c-axis ones. The parameters required to make the dimer-bond-operator theory reproduce the observed magnetic excitation energy are also very different to those required in the spin-wave treatment even though both are derived from Heisenberg models.

In contrast, the bilayer Hubbard model described in our work fits the data with more physically intuitive values. We employ a nearest-neighbour hopping ten times larger than the next-nearest-neighbour term, and a approximate one-to-one correspondence between intra- and interlayer hopping that is in line with the respective Ir-Ir bond lengths. The random phase approximation (RPA) also predicts the temperature dependence of the magnetic excitation satisfactorily (Fig. S5), providing evidence for the condensation of the excitonic longitudinal mode at T_N. This implies a close-by quantum critical point (QCP) which is indeed predicted by the theoretical model [9], therefore clarifying the microscopic nature of the material.

For completeness, we comment upon a further mechanism to generate magnetic longitudinal modes. This involves systems tuned to a QCP between states with spin 1 and spin 0 local moments in Mott insulators and can be realized by a subtle balance between non-cubic crystal field and moderate spin-orbit coupling (SOC) [10]. This intriguing state has been realized in Ca_2RuO_4 [11], but has minimal relevance to $Sr_3Ir_2O_7$ since it requires a d^4 electronic configuration.

2. MEAN FIELD AND RANDOM PHASE APPROXIMATIONS

We modeled the magnetic excitations of $Sr_3Ir_2O_7$ using a half-filled bilayer Hubbard Hamiltonian $\mathcal{H} = -\mathcal{H}_K + \mathcal{H}_I$, with $\mathcal{H}_I = U \sum_{\boldsymbol{r}} n_{\boldsymbol{r}\uparrow} n_{\boldsymbol{r}\downarrow}$ and

$$\mathcal{H}_K = \sum_{\boldsymbol{r},\boldsymbol{\delta}_\nu} t_\nu \boldsymbol{c}_{\boldsymbol{r}}^\dagger \boldsymbol{c}_{\boldsymbol{r}+\boldsymbol{\delta}_\nu} + t_z \sum_{\boldsymbol{r}_\perp} \boldsymbol{c}_{(\boldsymbol{r}_\perp,1)}^\dagger e^{i\frac{\alpha}{2}\epsilon_{\boldsymbol{r}}\sigma_z} \boldsymbol{c}_{(\boldsymbol{r}_\perp,2)} + \text{H.c.}, \tag{1}$$

where $t_\nu, t_z, \alpha, U \in \mathbb{R}$ as defined in the main text. The system has an easy z-axis spin anisotropy for $\alpha \neq 0$, and the ground state can have Néel ordering, $\langle S_{\boldsymbol{r}}^\mu \rangle = (-1)^{\gamma_{\boldsymbol{r}}} M \delta_{\mu z}$, where $\gamma_{\boldsymbol{r}} = (1 + \epsilon_{\boldsymbol{r}})/2$, $S_{\boldsymbol{r}}^\mu = 1/2 c_{\boldsymbol{r}}^\dagger \sigma^\mu c_{\boldsymbol{r}}$ $(\mu = x, y, z)$ and M is the magnetization. Following Ref. [9], a mean-field decoupling of $\mathcal{H}_I$ leads to $\mathcal{H}^{MF} = \sum_{\boldsymbol{k}\in BZ} \boldsymbol{c}_{\boldsymbol{k}}^\dagger \mathcal{H}_{\boldsymbol{k}}^{MF} \boldsymbol{c}_{\boldsymbol{k}}$ with

$$\mathcal{H}_{\boldsymbol{k}}^{MF} = \begin{pmatrix} \epsilon_{\boldsymbol{k}}^{(2)} + UM\sigma_z & \epsilon_{\boldsymbol{k}}^{(1)} - t_z \cos(k_z) e^{-i\frac{\alpha}{2}\sigma_z} \\ \epsilon_{\boldsymbol{k}}^{(1)} - t_z \cos(k_z) e^{i\frac{\alpha}{2}\sigma_z} & \epsilon_{\boldsymbol{k}}^{(2)} - UM\sigma_z \end{pmatrix}, \tag{2}$$

where $\boldsymbol{c}_{\boldsymbol{k}} \equiv (c_{\mathcal{A}\uparrow,\boldsymbol{k}}, c_{\mathcal{A}\downarrow,\boldsymbol{k}}, c_{\mathcal{B}\uparrow,\boldsymbol{k}}, c_{\mathcal{B}\downarrow,\boldsymbol{k}})^T$ is the Fourier transformed operator, and

$$\epsilon_{\boldsymbol{k}}^{(1)} = -2t_1 \left(\cos \frac{k_1 + k_2}{2} + \cos \frac{k_1 - k_2}{2} \right), \tag{3}$$

$$\epsilon_{\boldsymbol{k}}^{(2)} = -2t_2 \left(\cos k_1 + \cos k_2 \right), \tag{4}$$

and $\boldsymbol{k} \equiv (\boldsymbol{k}_\perp, k_z)$ a wavevector in the first Brillouin zone (BZ) *i.e.* $\boldsymbol{k}_\perp = k_1 \boldsymbol{b}_1' + k_2 \boldsymbol{b}_2'$, with $\boldsymbol{b}_1' = (1/2, -1/2)$, $\boldsymbol{b}_2' = (1/2, 1/2)$, $k_1, k_2 \in [0, 2\pi)$ and $k_z = 0, \pi$. $\mathcal{H}_{\boldsymbol{k}}^{MF}$ is diagonalized by a 4×4 unitary matrix $U(\boldsymbol{k})$, yielding

$$c_{\gamma\sigma,\boldsymbol{k}} = \sum_n U_{(\gamma\sigma),n}(\boldsymbol{k}) \psi_{n,\boldsymbol{k}}, \tag{5}$$

where $n \equiv (s, \sigma)$ with $s = \pm$, $\sigma = \uparrow, \downarrow$, and each column of $U(\boldsymbol{k})$ is an eigenvector of $\mathcal{H}_{\boldsymbol{k}}^{MF}$:

$$X_{s\uparrow}(\boldsymbol{k}) = \begin{pmatrix} x_{\uparrow\boldsymbol{k}} \sqrt{\frac{1+sz_{\uparrow\boldsymbol{k}}}{2}} \\ 0 \\ s\sqrt{\frac{1-sz_{\uparrow\boldsymbol{k}}}{2}} \\ 0 \end{pmatrix}, \quad X_{s\downarrow}(\boldsymbol{k}) = \begin{pmatrix} 0 \\ x_{\downarrow\boldsymbol{k}} \sqrt{\frac{1-sz_{\downarrow\boldsymbol{k}}}{2}} \\ 0 \\ s\sqrt{\frac{1+sz_{\downarrow\boldsymbol{k}}}{2}} \end{pmatrix}, \tag{6}$$

where

$$x_{\sigma\boldsymbol{k}}=\frac{b_{\sigma\boldsymbol{k}}}{|b_{\sigma\boldsymbol{k}}|},\quad z_{\sigma\boldsymbol{k}}=\frac{\delta}{\sqrt{\delta^2+|b_{\sigma\boldsymbol{k}}|^2}},\; b_{\sigma\boldsymbol{k}}=\epsilon^{(1)}_{\boldsymbol{k}}-t_z\cos(k_z)e^{-i\sigma\frac{\alpha}{2}} \tag{7}$$

and $\delta=UM$. The corresponding eigenenergy, $\varepsilon_{s\sigma}(\boldsymbol{k})=\epsilon^{(2)}_{\boldsymbol{k}}+s\sqrt{\delta^2+|b_{\sigma\boldsymbol{k}}|^2}$, is independent of the spin flavour, $|b_{\sigma\boldsymbol{k}}|^2=b^2_{\boldsymbol{k}}$ and $z_{\sigma\boldsymbol{k}}\equiv z_{\boldsymbol{k}}$. The order parameter is determined by solving the self-consistent equation:

$$\begin{aligned}(-1)^{\gamma}M&=\frac{1}{\mathcal{N}_u}\sum_{\boldsymbol{r}\in\gamma}\langle S^z_{\boldsymbol{r}}\rangle\\&=\frac{1}{2\mathcal{N}_u}\sum_{\boldsymbol{r}\in\gamma}\sum_{\sigma}\sigma\langle c^{\dagger}_{\gamma\sigma,\boldsymbol{r}}c_{\gamma\sigma,\boldsymbol{r}}\rangle\\&=\frac{1}{2\mathcal{N}_u}\sum_{\boldsymbol{k}}\sum_{\sigma}\sigma\langle c^{\dagger}_{\gamma\sigma,\boldsymbol{k}}c_{\gamma\sigma,\boldsymbol{k}}\rangle\\&=\frac{1}{2\mathcal{N}_u}\sum_{\boldsymbol{k}}\sum_{\sigma}\sigma\sum_{n}\left|U_{(\gamma\sigma),n}(\boldsymbol{k})\right|^2 f_{n\boldsymbol{k}}\\&=(-1)^{\gamma}\frac{1}{2\mathcal{N}_u}\sum_{\boldsymbol{k}}z_{\boldsymbol{k}}\left(f_{-\boldsymbol{k}}-f_{+\boldsymbol{k}}\right),\end{aligned}$$

where $\mathcal{N}_u$ is the number of unit cells and $f_{s\boldsymbol{k}}=\frac{1}{1+e^{\beta(\varepsilon_s(\boldsymbol{k})-\mu)}}$ is the Fermi distribution function. We found the self-consistent solution δ and μ under the condition the electron density satisfies

$$2n=\frac{1}{2N_u}\sum_{\boldsymbol{k},n}f_{n\boldsymbol{k}}, \tag{8}$$

where n is the electron density of the system. At half-filling ($n=1/2$) and zero temperature, the critical value of U can be obtained by

$$\frac{1}{U_{\mathrm{c}}}=\frac{1}{2\mathcal{N}_u}\sum_{\boldsymbol{k}}\frac{\partial}{\partial\delta}z_{\boldsymbol{k}}\bigg|_{\delta=0}=\frac{1}{2\mathcal{N}_u}\sum_{\boldsymbol{k}}\frac{1}{|b_{\boldsymbol{k}}|}. \tag{9}$$

In the ordered phase ($U>U_{\mathrm{c}}$), the critical temperature is obtained by solving the equation

$$\frac{1}{U}=\frac{1}{2\mathcal{N}_u}\sum_{\boldsymbol{k}}\frac{\partial}{\partial\delta}\left(z_{\boldsymbol{k}}\left(f_{-\boldsymbol{k}}-f_{+\boldsymbol{k}}\right)\right)\bigg|_{\delta=0}=\frac{1}{2\mathcal{N}_u}\sum_{\boldsymbol{k}}\frac{f_{-\boldsymbol{k}}-f_{+\boldsymbol{k}}}{|b_{\boldsymbol{k}}|} \tag{10}$$

together with Eq. (8) for $\delta=0$. The magnetic susceptibilities of the transverse and the longitudinal modes within the RPA are given by

$$\chi^{+-}(\boldsymbol{q},i\omega_n)=\frac{1}{\tau^0-U\chi^{+-}_0(\boldsymbol{q},i\omega_n)}\chi^{+-}_0(\boldsymbol{q},i\omega_n), \tag{11}$$

$$\chi^{zz}(\boldsymbol{q},i\omega_n)=\frac{1}{\tau^0-\frac{U}{2}\chi^{zz}_0(\boldsymbol{q},i\omega_n)}\chi^{zz}_0(\boldsymbol{q},i\omega_n), \tag{12}$$

respectively, where τ_0 is the 2×2 identity matrix. Here χ^{+-} and χ^{zz} are 2×2 matrices in the sublattice space, while χ^{+-}_0 and χ^{zz}_0 are the bare magnetic susceptibilities [9]. In the calculations of the spectra shown in Fig. S5, we set the broadening factor to $\eta=10^{-4}$ eV. The dynamical spin structure factors in Figs. 3c and d of the main manuscript are shown after convolution with the experimental resolution.

3. ILLUSTRATION OF THE EXCITON

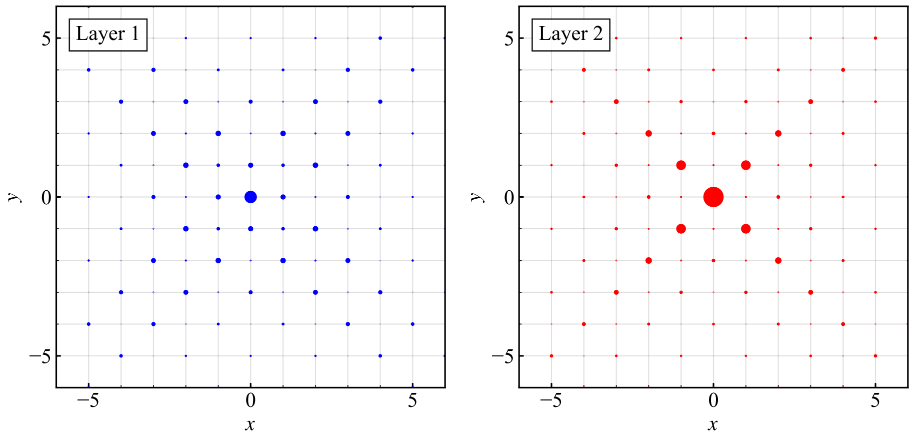

FIG. S1. **Illustration of the exciton.** Left and right panels show the real-space Ir lattice for the two layers that make up the $Sr_3Ir_2O_7$ bilayer structure. The area of the circles at each lattice point is proportional to the modulus squared of the exciton wavefunction under the condition that a spin down hole is fixed at the origin of layer 1. The left panel shows an enhanced probability of a spin-down electron surrounding the reference hole in layer 1, and the right panel shows an enhanced occupation living above the hole in layer 2. x and y represent the two orthogonal direction within the bilayer in units of the Ir-Ir spacing.

In an antiferromagnetic excitonic insulator, the relevant bound electron-hole pairs are magnetic modes that are not directly visible to charge-sensitive spectral probes. We can, however, plot the spatial distribution of the electron and hole to illustrate their real space configuration, based on our theory, which is validated by its close agreement with our measurements of the magnetic dispersion and the observed charge gap. As seen in Fig. S1 the exciton is made up of a specific spin and charge configuration distributed between the two layers and dispersed over a few lattice constant laterally. In Fig. S1 the state is represented in terms of electron/hole population with respect to the average band filling of 1 electron per orbital, as pairing between spin-down holes with spin-down electrons, which is equivalent to pairing electrons with opposite spin.

4. EXCITONIC LONGITUDINAL MODE CONDENSATION AT T_{N}

The involvement of the excitonic longitudinal mode in the formation of ground-state magnetism was studied using thermal fluctuations. Figures 4a-d of the main manuscript show the temperature dependence of the magnetic excitation spectra at (0, 0) and $(0.5, 0.5)$ for $q_c = 0$ and 0.5, which were generated from temperature dependent energy-loss spectra such as shown in Fig. S2.

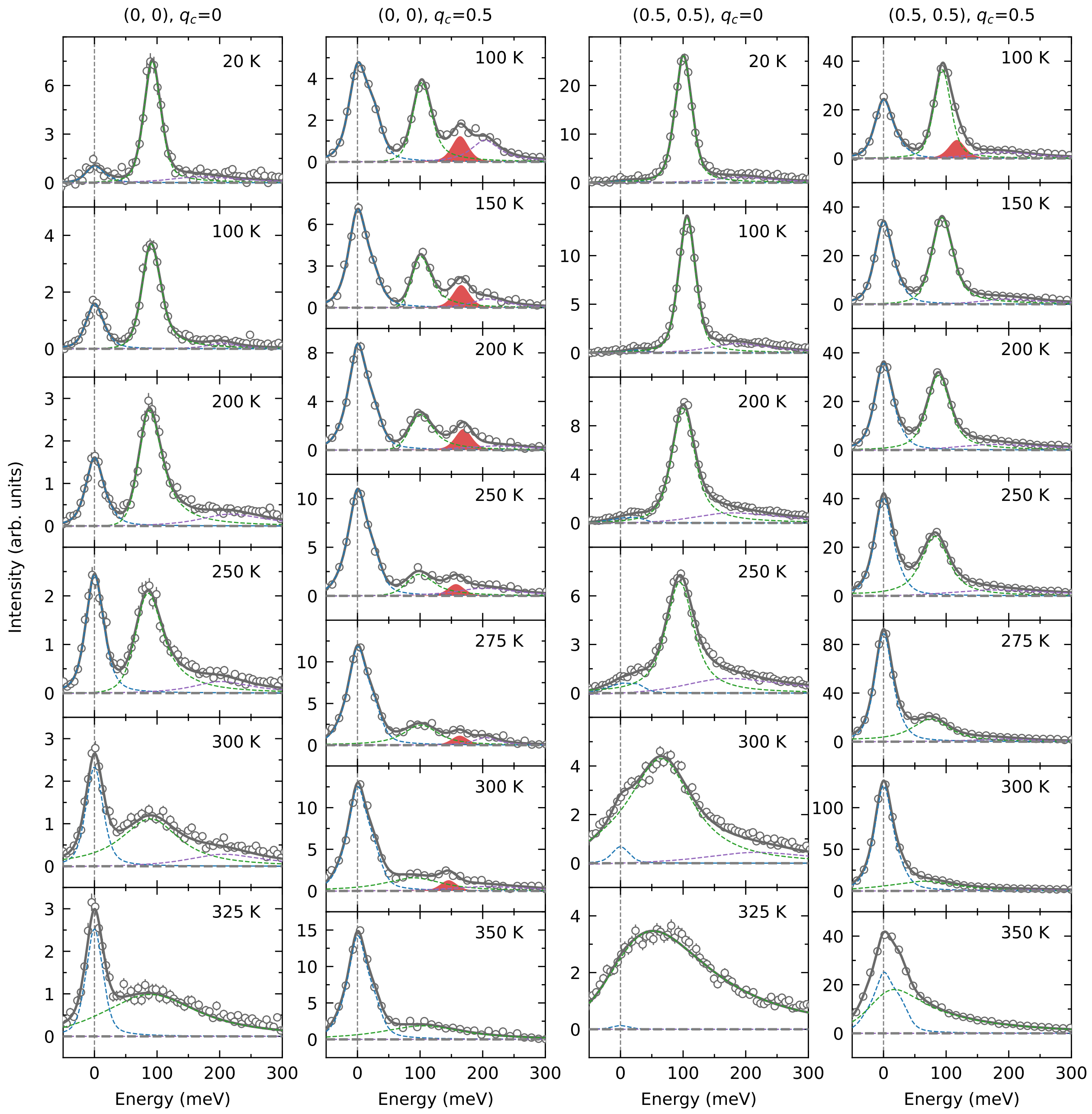

FIG. S2. **Temperature dependence of energy-loss spectra at (0, 0) and (0.5, 0.5).** RIXS spectra at (0, 0) and (0.5, 0.5) with $q_c = 0$ and 0.5 for specific temperatures. The black circles represent the data and dotted lines outline the different components of the spectrum, which are summed to produce the solid line representing the total spectrum. The fitting procedure is described in detail in the Methods section of the main manuscript. Error bars are determined using Possonian statistics. The results support the longitudinal mode condensation at T_{N}.

The data reveal a substantial softening of the excitonic longitudinal mode, while only minimal detectable softening is observed in the transverse channel. This is supported by further temperature dependent energy-loss spectra at $(0.5, 0)$ and $(0.25, 0.25)$ shown in Figs. S3a-d. In agreement with the temperature-dependence of the quasi-elastic intensity at (0, 0) (Fig. 4e in the main manuscript), a monotonic enhancement is also observed at $(0.5, 0)$ and $(0.25, 0.25)$ (Figs. S3e-f).

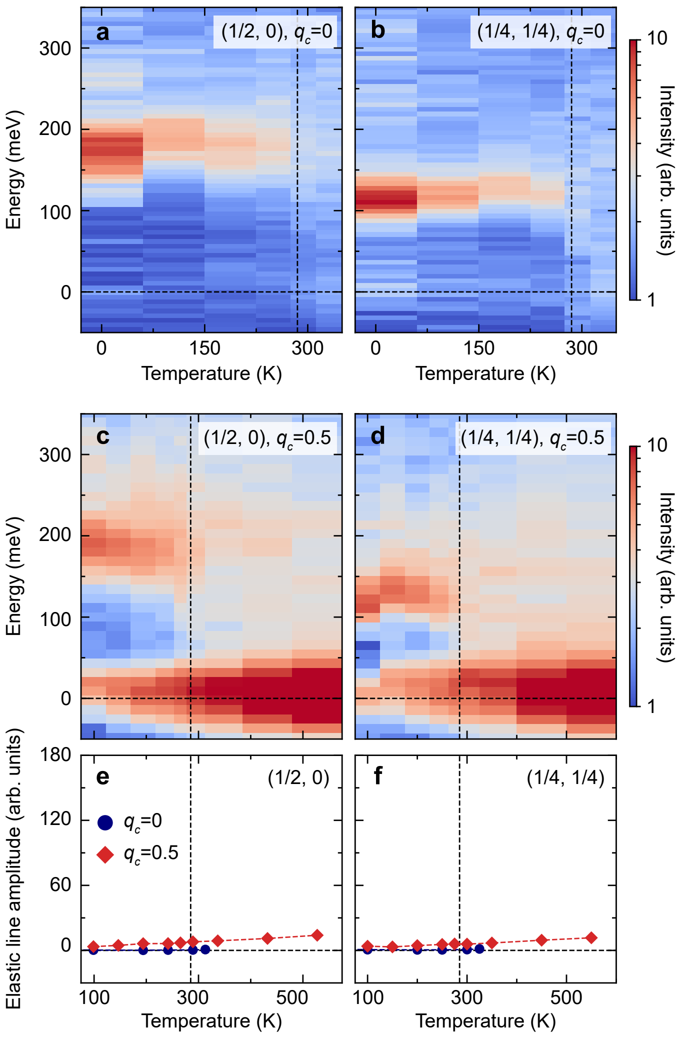

FIG. S3. **Additional temperature-dependent energy-loss spectra. a-d** Temperature dependence of the $Sr_3Ir_2O_7$ excitation spectrum at $(0.5, 0)$ and $(0.25, 0.25)$ for $q_c = 0$ and 0.5. The color bar indicates the strength of the magnetic excitations. **e, f** Quasi-elastic intensity as function of temperature for $q_c = 0$ and 0.5 in blue and red, respectively. The data reveal the expected monotonic enhancement of the quasi-elastic intensity with increasing temperature.

As explained in the main text, increasing temperature broadens the spectra considerably, so we leverage the symmetry properties of the modes to unveil the detailed mode behavior. The transverse mode energy is predicted to be independent of q_c and we can study this mode in isolation at $q_c = 0$ and see that it shows minimal softening. At $q_c = 0.5$, however, the transverse and longitudinal modes are present and considerable softening is observed, but since the transverse mode has minimal softening, we can assign this softening to arise predominately from the longitudinal mode. Although the damping effect of the excitonic longitudinal mode makes it difficult to directly attribute the softening to the longitudinal mode, a mode condensation at (0.5, 0.5) $q_c = 0.5$ is further supported by the enhancement of the low-energy excitation spectrum (0.5, 0.5) $q_c = 0.5$ that is absent at $q_c = 0$ (see Fig. S4).

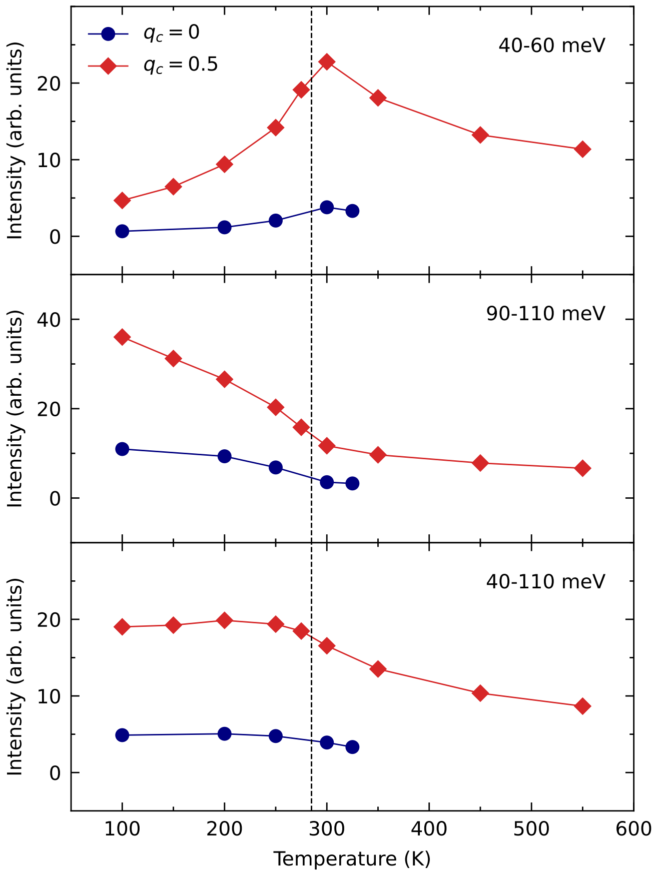

FIG. S4. **Temperature dependence of the low-energy excitation spectrum. a-d** Temperature dependence of the low-energy excitation spectrum integrated over an energy range of 40-60, 90-110 and 40-110 meV at (0.5, 0.5) $q_c = 0$ and 0.5. Although the damping effect of the excitonic longitudinal mode makes it difficult to directly attribute the softening to the longitudinal mode, a mode condensation at (0.5, 0.5) $q_c = 0.5$ is further supported by the enhancement of the low-energy quasi-elastic intensity.

The temperature dependence of the transverse and longitudinal magnetic excitation spectrum was calculated using a RPA in the thermodynamic limit (the Hubbard Hamiltonian is described in the Methods section of the main manuscript). Figure S5 displays the predicted dynamic structure factor at $q_c = 0$ and 0.5 for $T = 20$, 200, 250, 275, 300 and 550 K. The color bar indicates the strength of the magnetic excitations. The results show that upon increasing temperature the transverse mode has only minimal detectable softening, which is expected in view of the Ising nature of magnetism. This is in strong contrast with the temperature dependence of the longitudinal mode at $q_c = 0.5$. At $T = 20$ K the exctonic longitudinal mode is well-defined around (0.5, 0.5) and (0, 0), and dissolves into the electron-hole continuum at wavevetors away from these reciprocal-lattice positions. Increasing temperature yields a continuous softening and ultimately to the condensation of the mode at $T_{\rm N} = 285$ K. This shows that the excitonic longitudinal mode establishes the magnetic long-range order in $Sr_3Ir_2O_7$.

Above $T_{\rm N}$ our calculations reveal remnant longitudinal intensity, which can be assigned to preformed exciton pairs. This classifies the phase transition as a Bose-Einstein condensation, opposed to Bardeen-Cooper-Schrieffer-like transition. The Bose-Einstein nature can be traced back to the SOC of the theoretical model shown in the Methods section of the main manuscript. The finite SOC included in the interlayer hopping establishes a narrow-band insulator even at $U = 0$. Thus, preformed excitons exist in the paramagnetic state at $U < U_c$, and $U > U_c$ for $T > T_{\rm N}$.

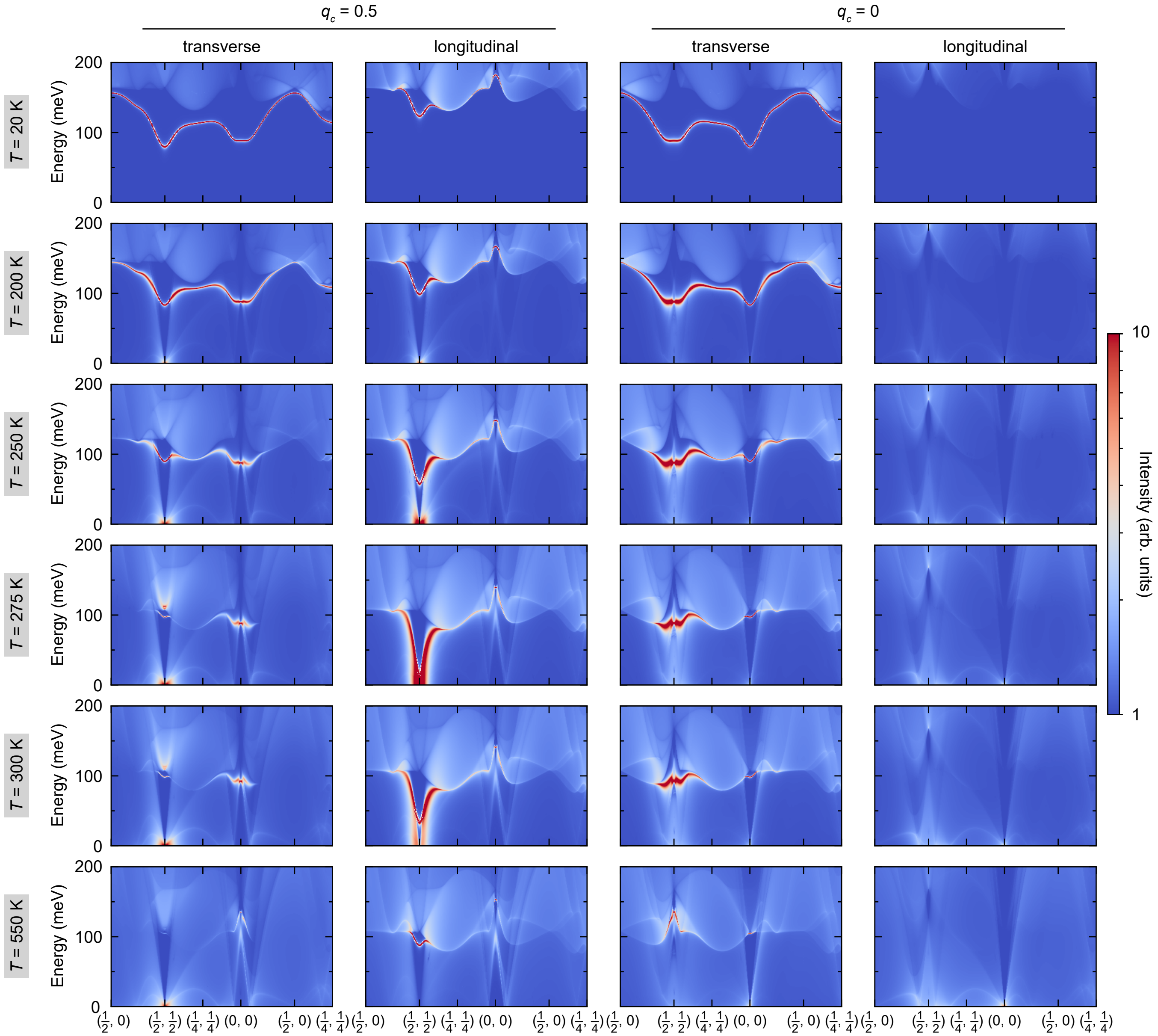

FIG. S5. **Theoretical prediction of excitonic mode condensation at $T_{\rm N}$.** Theoretical calculation of the transverse and longitudinal magnetic excitation spectrum at $q_c = 0$ and 0.5 as function of temperature. The color bar indicates the strength of the magnetic excitations.

5. MINIMAL CONTRIBUTION OF CRITICAL SCATTERING TO QUASI-ELASTIC INTENSITY

In the main manuscript, we interpret the temperature-dependent RIXS spectra in terms of longitudinal mode softening. Another possible source of quasi-elastic scattering is critical scattering associated with the sample's Néel order. As mentioned in the main text, the bilayer separation in $Sr_3Ir_2O_7$ is incommensurate with the c-axis constant, so critical scattering from Néel order and quasi-elastic scattering from longitudinal mode softening can be distinguished based on their c-axis momentum dependence. We plot the q_c-dependence of the $(0.5, 0.5)$ quasi-elastic line intensity at two temperatures in the vicinity of $T_{\rm N}$ a function of q_c in Fig. S6. The results show that longitudinal mode softening dominates the measured intensity.

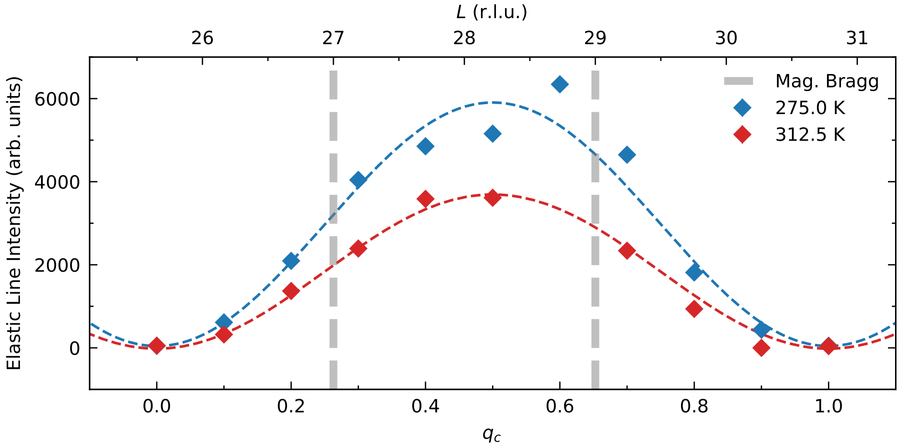

FIG. S6. **Origin of quasi-elastic intensity near T_{N}.** The c-axis momentum dependence of the quasi-elastic intensity at $(0.5, 0.5)$ is plotted at two temperatures near T_{N}. The overall tend in intensity follows a $\sin^2(\pi q_c)$ dependence illustrated by the dotted line, as expected for intensity that arises from longitudinal mode softening. This is quite distinct from critical scattering from Néel order, which would be centered around the magnetic Bragg peaks shown as vertical gray lines.

6. ORBITAL CURRENTS

As was predicted in Ref. [12], antiferromagnetic order in an excitonic insulator with finite SOC could in principle induce orbital antiferromagnetism associated with charge currents on plaquettes of the lattice. However, we have verified that this does not occur in $Sr_3Ir_2O_7$ by calculating the expectation value of the current operator around a plaquette. The only source of orbital antiferromagnetism in $Sr_3Ir_2O_7$ is the strong intra-atomic SOC that leads to a mixed spin-orbital character of the atomic magnetic moments.

[1] Kim, J. *et al.* Large spin-wave energy gap in the bilayer iridate $Sr_3Ir_2O_7$: Evidence for enhanced dipolar interactions near the Mott metal-insulator transition. *Phys. Rev. Lett.* **109**, 157402 (2012).

[2] Moretti Sala, M. *et al.* Evidence of quantum dimer excitations in $Sr_3Ir_2O_7$. *Phys. Rev. B* **92**, 024405 (2015).

[3] Gretarsson, H. *et al.* Two-magnon raman scattering and pseudospin-lattice interactions in Sr_2IrO_4 and $Sr_3Ir_2O_7$. *Phys. Rev. Lett.* **116**, 136401 (2016).

[4] Hogan, T. *et al.* Disordered dimer state in electron-doped $Sr_3Ir_2O_7$. *Phys. Rev. B* **94**, 100401 (2016).

[5] Li, S. *et al.* Symmetry-resolved two-magnon excitations in a strong spin-orbit-coupled bilayer antiferromagnet. *Phys. Rev. Lett.* **125**, 087202 (2020).

[6] Carter, J.-M., Shankar V., V. & Kee, H.-Y. Theory of metal-insulator transition in the family of perovskite iridium oxides. *Phys. Rev. B* **88**, 035111 (2013).

[7] Igarashi, J.-i. & Nagao, T. Analysis of resonant inelastic x-ray scattering from Sr_2IrO_4 in an itinerant-electron approach. *Phys. Rev. B* **90**, 064402 (2014).

[8] Fujita, M., Ikeuchi, K., Kajimoto, R. & Nakamura, M. Magnetic excitations of $Sr_3Ir_2O_7$ observed by inelastic neutron scattering technique. *Journal of the Physical Society of Japan* **90**, 025001 (2021).

[9] Suwa, H., Zhang, S.-S. & Batista, C. D. Exciton condensation in bilayer spin-orbit insulator. *Phys. Rev. Research* **3**, 013224 (2021).

[10] Khaliullin, G. Excitonic magnetism in Van Vleck–type d^4 Mott insulators. *Phys. Rev. Lett.* **111**, 197201 (2013).

[11] Jain, A. *et al.* Higgs mode and its decay in a two-dimensional antiferromagnet. *Nature Physics* **13**, 633–637 (2017).

[12] Halperin, B. I. & Rice, T. M. Possible anomalies at a semimetal-semiconductor transistion. *Rev. Mod. Phys.* **40**, 755–766 (1968).
